# TRPS1 Expression Is Frequently Seen in a Subset of Cutaneous Mesenchymal Neoplasms and Tumors of Uncertain Differentiation: A Potential Diagnostic Pitfall

**DOI:** 10.3390/dermatopathology11030021

**Published:** 2024-07-15

**Authors:** Moon Joo Kim, Yi A. Liu, Yunyi Wang, Jing Ning, Woo Cheal Cho

**Affiliations:** 1 Department of Anatomic Pathology, The University of Texas MD Anderson Cancer Center, Houston, TX 77030, USA; moonjoo0801@gmail.com; 2 Department of Pathology and Laboratory Medicine, Royal Columbian Hospital, University of British Columbia, New Westminster, BC V3L 3W7, Canada; yiariel.liu@fraserhealth.ca; 3 Department of Biostatistics, The University of Texas MD Anderson Cancer Center, Houston, TX 77030, USA; ywang76@mdanderson.org (Y.W.); jning@mdanderson.org (J.N.)

**Keywords:** TRPS1, immunohistochemistry, cutaneous mesenchymal neoplasms, cutaneous tumors of uncertain differentiation, atypical fibroxanthoma

## Abstract

Although extensively studied in cutaneous epithelial neoplasms, the TRPS1 immunoreactivity in cutaneous mesenchymal neoplasms and tumors of uncertain differentiation (CMNTUDs), such as atypical fibroxanthoma (AFX), remains largely unexplored. We assessed TRPS1 immunoreactivity in 135 CMNTUDs, comprising 46 fibrohistiocytic/fibroblastic tumors, 28 vascular tumors, 24 peripheral nerve sheath tumors (PNSTs), 21 tumors of uncertain differentiation, and 16 smooth muscle tumors. Additionally, we included selected cases of melanoma with spindled cell morphology or desmoplastic features (n = 9) and sarcomatoid squamous cell carcinoma (SSCC) (n = 5) to compare TRPS1 expression patterns with those of AFX. TRPS1 expression was prevalent in dermatofibromas (24/24), leiomyomas (8/8), AFXs/pleomorphic dermal sarcoma (PDS) (20/21), dermatofibrosarcomas protuberans (14/22), and leiomyosarcomas (6/8). It was uncommon in angiosarcomas (3/20), Kaposi sarcomas (2/8), and neurofibromas (5/17) and absent in perineuriomas (0/2). AFXs/PDS exhibited the highest median H-score of 240, contrasting with minimal TRPS1 immunoreactivity in vascular neoplasms and PNSTs, with median H-scores consistently below 10. Significant differences in H-score were observed between AFXs/PDS and angiosarcomas (*p* < 0.001), melanomas (*p* < 0.001), and leiomyosarcomas (*p* = 0.029). However, no significant difference was found compared to SSCCs, suggesting limited discriminatory power of TRPS1 in this context. This study sheds light on TRPS1 expression patterns in a subset of CMNTUDs, extending beyond prior studies primarily focused on epithelial tumors, while underscoring potential pitfalls associated with TRPS1 immunohistochemistry.

## 1. Introduction

TRPS1 immunohistochemistry (IHC) has surged in popularity among surgical pathologists in recent years, initially believed to offer high sensitivity and specificity for carcinomas of mammary origin [1]. However, recent studies challenge this notion, revealing that TRPS1 expression extends beyond breast neoplasms. Robust TRPS1 expression has been observed in various cutaneous epithelial neoplasms, including squamous cell carcinomas [2,3], mammary and extramammary Paget diseases [2,4], and adnexal neoplasms [3,5,6,7]. In dermatopathology, TRPS1 emerges as a valuable tool, particularly in distinguishing primary from secondary extramammary Paget disease, especially when arising in non-perianal cutaneous sites [4]. Moreover, TRPS1 aids in confirming diagnoses of endocrine mucin-producing sweat gland carcinoma (EMPSGC) when combined with neuroendocrine markers, notably INSM1, as no other morphologic mimics of EMPSGC demonstrate strong co-expression of TRPS1 and INSM1 [5].

While TRPS1 expression patterns have been extensively studied in cutaneous epithelial neoplasms, data regarding its expression in cutaneous mesenchymal neoplasms and tumors of uncertain differentiation, such as atypical fibroxanthoma (AFX), remain limited. In this study, our objective was to assess the immunohistochemical expression patterns of TRPS1 in a subset of mesenchymal neoplasms and tumors of uncertain differentiation originating in the skin. We aimed to evaluate its potential diagnostic utility and identify any pitfalls associated with its application in these cutaneous neoplasms.

## 2. Materials and Methods

### 2.1. Case Selection

Cases of cutaneous mesenchymal neoplasms and tumors with uncertain differentiation were identified from pathology archives with approval from the Institutional Review Board of the University of Texas MD Anderson Cancer Center (protocol 2022-0662), along with a waiver of informed consent. A preliminary review of H&E-stained slides was conducted to identify potential cases before retrieving corresponding formalin-fixed, paraffin-embedded (FFPE) tissue blocks, ensuring adequate lesional cells for subsequent immunohistochemical analysis. From this collection, a total of 135 cases were selected for the study, comprising 20 cases of AFX, 1 case of pleomorphic dermal sarcoma (PDS), 20 cases of angiosarcoma, 8 cases of Kaposi sarcoma, 24 cases of dermatofibroma (DF), 22 cases of dermatofibrosarcoma protuberans (DFSP), 22 cases of neurofibroma (NF), 2 cases of perineurioma, 8 cases of leiomyoma, and 8 cases of leiomyosarcoma (Table 1). Furthermore, several representative cases of melanoma (9), primarily exhibiting spindled cell morphology or desmoplastic features, and sarcomatoid squamous cell carcinoma (SCC) (5) were included to serve as morphological differentials of AFX for comparative analysis of TRPS1 expression. Of note, the single case of PDS was distinguished from the other 20 cases of AFX in our study by its extensive involvement of deeper tissues, such as the subcutis.

### 2.2. Immunohistochemical Analysis

A 4–5 µm thick paraffin section was freshly cut from each selected case’s FFPE tissue block. The unstained slides underwent immunohistochemical analysis using a monoclonal anti-TRPS1 rabbit anti-human antibody (Abcam, EPR16171, 1:2000) with a Leica Bond Max autostainer system (Leica Biosystems, GmbH, Nussloch, Germany) following standard automated protocols. Positive immunoreactivity was determined by nuclear expression of TRPS1 in tumor cells. TRPS1 expression intensity was categorized into four levels (none, 0; weak, 1+; moderate, 2+; strong, 3+), with innate eccrine glands serving as the internal control for 3+ intensity (strong). H-scores were calculated by multiplying the percentage of positive tumor cells by the corresponding TRPS1 expression intensity, resulting in total scores ranging from 0 to 300. The IHC results were independently reviewed by three board-certified pathologists (M.J.K., Y.A.L., and W.C.C.), including two dermatopathologists (Y.A.L. and W.C.C.).

### 2.3. Statistical Analysis

The expression characteristics of TRPS1 in tumors were summarized using descriptive statistics. Continuous variables were presented using mean, median, interquartile range, and range, while categorical variables were described using frequency and proportion (%). To compare these variables among overall groups, analysis of variance (ANOVA) and the Kruskal–Wallis rank sum test were utilized for continuous variables, whereas Fisher’s exact test was employed for categorical variables. Pairwise comparisons were conducted using the two-sample *t*-test and Wilcoxon rank sum test. The normality assumption necessary for the ANOVA and *t*-test was assessed. A *p*-value of less than 0.05 was considered statistically significant. All statistical analyses were conducted using R version 4.2.3 (R Foundation for Statistical Computing, Vienna, Austria). Of note, the single case of PDS was merged with the 20 cases of AFX during statistical analysis due to their morphological similarities. Additionally, two cases of perineurioma were excluded from the analysis due to their small sample size.

## 3. Results

### 3.1. TRPS1 Expression in Cutaneous Mesenchymal Neoplasms

**Figure 1:**
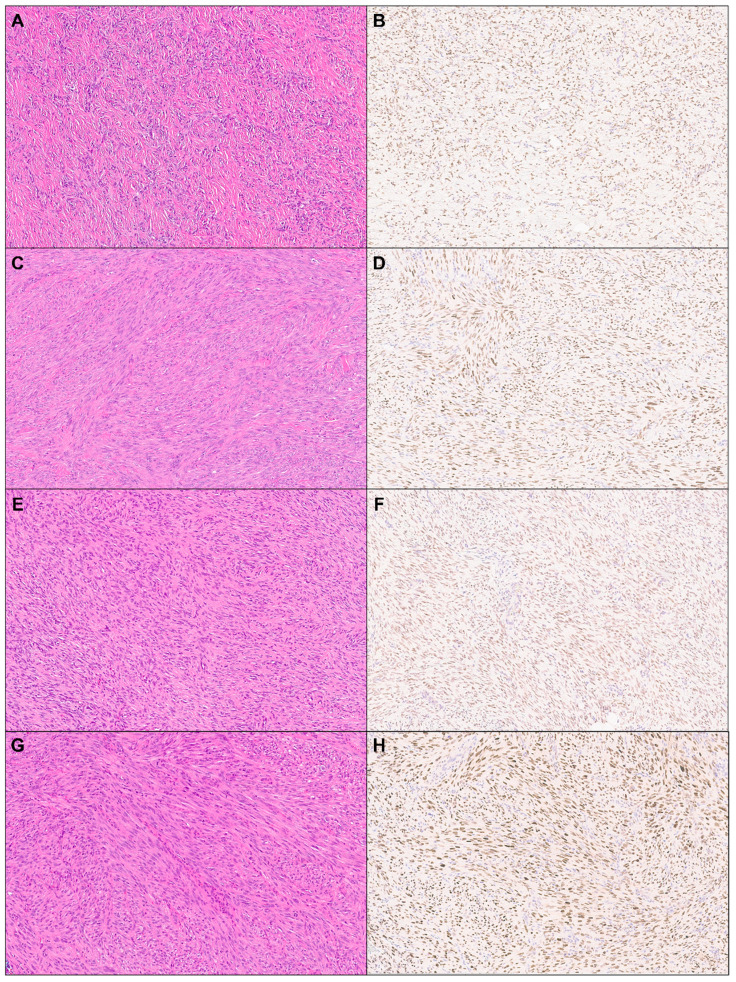
TRPS1 expression in cutaneous mesenchymal neoplasms and tumors of uncertain differentiation. Representative cases of dermatofibroma ((**A**): H&E ×10, (**B**): TRPS1 ×10), leiomyoma ((**C**): H&E ×10, (**D**): TRPS1 ×10), dermatofibrosarcoma protuberans ((**E**): H&E ×10, (**F**): TRPS1 ×10), and leiomyosarcoma ((**G**): H&E ×10, (**H**): TRPS1 ×10).

**Figure 2:**
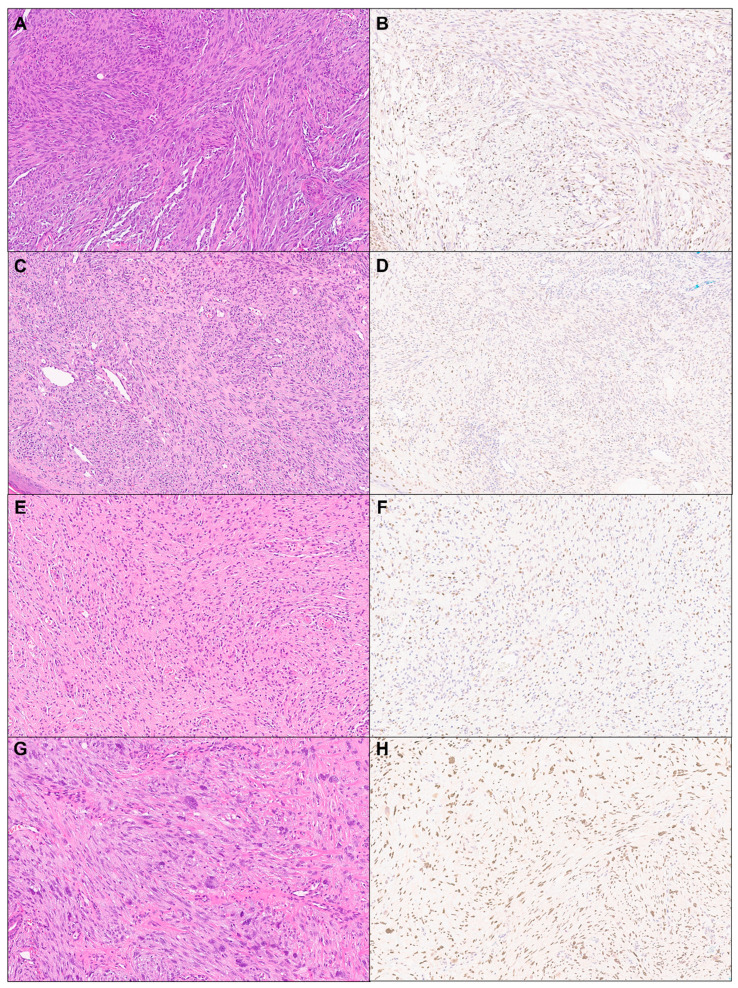
TRPS1 expression in cutaneous mesenchymal neoplasms and tumors of uncertain differentiation. Representative cases of angiosarcoma ((**A**): H&E ×10, (**B**): TRPS1 ×10), Kaposi sarcoma ((**C**): H&E ×10, (**D**): TRPS1 ×10), neurofibroma ((**E**): H&E ×10, (**F**): TRPS1 ×10), and atypical fibroxanthoma ((**G**): H&E ×10, (**H**): TRPS1 ×10).

A total of 114 cases of cutaneous mesenchymal neoplasms were included in the analysis (Table 1). TRPS1 expression was consistently observed in DFs (100%; 24/24) and leiomyomas (100%; 8/8) (Figure 1). It was also frequently detected in DFSPs (63.6%; 14/22) and leiomyosarcomas (75%; 6/8) (Figure 1). In contrast, TRPS1 expression was less common in angiosarcomas (15.0%; 3/20), Kaposi sarcomas (25.0%; 2/8), and neurofibromas (22.7%; 5/17) (Figure 2). The two cases of perineuriomas showed an absence of TRPS1 expression.

The overall difference in H-score across all tumor types tested was statistically significant (*p* < 0.001; Table 1). The median H-score for TRPS1 expression was highest in leiomyomas (170) followed by DFs (140). In contrast, the median H-scores in their malignant counterparts, leiomyosarcomas and DFSPs, were lower than 100. Notably, when conducting pairwise comparisons, the difference in H-score between DFs and DFSPs was statistically significant (*p* < 0.001; Supplemental Table S1), whereas no significant difference in H-score was seen between leiomyomas and leiomyosarcomas (*p* = 0.205; Supplemental Table S2).

### 3.2. TRPS1 Expression in Cutaneous Tumors with Uncertain Differentiation

Almost all cases of AFX/PDS (95.2%; 20/21) demonstrated TRPS1 immunoreactivity, with their expression predominantly at least moderate in intensity (90%; 18/20), yielding a median H-score of 240 (Figure 2 and Table 1). The single case of PDS exhibited diffuse and strong TRPS1 immunoreactivity, with an H-score of 285.

### 3.3. Comparative Analysis of TRPS1 Expression in Atypical Fibroxanthoma/Pleomorphic Dermal Sarcoma Group and Their Morphological Mimics

Given that sarcomatoid SCCs, melanomas, leiomyosarcomas, and angiosarcomas are frequently considered in the morphological differential diagnosis of AFX, the TRPS1 expression characteristics of the AFX/PDS group were compared with those of the former three entities. The difference in intensity, proportion, and H-score for TRPS1 expression between AFXs/PDS and angiosarcomas was statistically significant (*p* < 0.001; Table 2). Similarly, a significant difference was observed between AFXs/PDS and melanomas (*p* < 0.001; Table 3). However, while there was a statistically significant difference in H-score between AFXs/PDS and leiomyosarcomas (*p* = 0.029; Supplemental Table S3), no differences were found in TRPS1 expression intensity and proportion between the two groups (Supplemental Table S3). Comparing with sarcomatoid SCCs, AFXs/PDS showed no significant difference in terms of TRPS1 expression intensity, proportion, and H-score (Supplemental Table S4).

## 4. Discussion

Previous studies have primarily focused on examining TRPS1 expression patterns in cutaneous epithelial and melanocytic tumors [2,3,4,5]. However, to our knowledge, there has been limited investigation into the TRPS1 immunoreactivity status in cutaneous mesenchymal tumors and tumors of uncertain differentiation, such as AFXs or PDSs. Our research fills this gap by demonstrating that strong and diffuse TRPS1 expression is not exclusive to certain cutaneous epithelial neoplasms, such as SCCs [2,3], mammary and extramammary Paget diseases [2,4], and EMPSGCs [5], but is also observed in a subset of non-epithelial and non-melanocytic cutaneous neoplasms. In our study, we found that TRPS1 expression was frequently observed in tumors of uncertain differentiation (AFX and PDS), tumors of fibrohistiocytic origin (DF and DFSP), and tumors of smooth muscle origin (leiomyoma and leiomyosarcoma). Notably, the AFX/PDS group exhibited the highest median H-score of 240 (Table 1). Conversely, TPRS1 expression was rare and minimal, if present at all, in cutaneous vascular neoplasms (angiosarcoma and Kaposi sarcomas) and peripheral nerve sheath tumors (NF), with their median H-scores consistently less than 10 (Table 1).

Intrigued by these findings, particularly the frequent expression of TRPS1 in the AFX/PDS group, we extended our investigation to include morphological mimics, such as sarcomatoid SCCs and melanomas, especially those exhibiting spindled cell morphology or desmoplastic features. Our aim was to assess whether TRPS1 could serve as a diagnostic tool in distinguishing these tumors. Our findings indicate that the presence of strong and diffuse TRPS1 expression may lean towards a diagnosis of AFX over angiosarcoma or melanoma, provided it is supported by other commonly used discriminatory immunohistochemical markers like ERG and SOX10. However, the utility of TRPS1 in distinguishing AFXs from leiomyosarcomas or sarcomatoid SCCs seems to be limited, particularly with the latter group. Recent studies have highlighted the frequent expression of TRPS1 in SCCs [2,3], suggesting that its presence in sarcomatoid SCCs may not be entirely coincidental. However, these prior studies mainly focused on well- and moderately differentiated SCCs [2,3], leaving the TRPS1 immunoreactivity status in sarcomatoid SCCs largely unexplored. Nevertheless, our data imply that relying solely on TRPS1 for the diagnosis of AFXs and their histopathologic mimics should be cautioned against. Lastly, while primary synovial sarcoma (SS) rarely involves the superficial portions of the skin, it may also be considered in the differential diagnosis of AFXs based on morphological similarities. Our study did not include this tumor type in our cohort; however, a recent study emphasized that TRPS1 is frequently expressed in SSs [8]. According to this research, TRPS1 immunoreactivity was observed in up to 86% of SS cases, with approximately 30% showing diffuse and intense TRPS1 expression [8].

It is worth noting that while our study may represent the first extensive immunohistochemical analysis of TRPS1 specifically targeting mesenchymal tumors or tumors of uncertain differentiation in the skin, initial documentation of TRPS1 immunohistochemical expression in non-epithelial and non-melanocytic skin cells occurred in reactive fibroblasts/myofibroblasts within dermal granulation tissues and scars [9]. This observation was highlighted in a case study revealing an “unexpected” expression of TRPS1 in dermal granulation tissues/scars of breast skin in a patient with a history of bilateral breast carcinomas, underscoring an important pitfall associated with the use of TRPS1 IHC [9]. Subsequently, as briefly mentioned above, a study demonstrated frequent TRPS1 expression in SSs [8], a type of mesenchymal tumor with uncertain differentiation. Therefore, the presence of TRPS1 expression in certain fibrohistiocytic and/or fibroblastic tumors, such as DFs and DFSPs, and AFXs observed in our study is not merely coincidental.

A limitation of our study is the small cohort size and the absence of a complete list of other cutaneous mesenchymal tumors. Further investigations with larger and more comprehensive cohorts are necessary to fully extrapolate the findings of TRPS1 immunoreactivity observed in various cutaneous neoplasms from both our current study and prior research. Given that the majority of published studies on TRPS1 immunohistochemical expression have predominantly focused on epithelial tumors, the aim of our study was to bring attention to a potential pitfall associated with the use of TRPS1 IHC in cutaneous mesenchymal tumors and tumors of uncertain differentiation frequently encountered in routine dermatopathology practice.

In conclusion, our study has provided novel insights into the TRPS1 expression patterns in a subset of cutaneous mesenchymal tumors and tumors of uncertain differentiation. By extending beyond previous research primarily focused on epithelial neoplasms, we have highlighted the importance of recognizing potential pitfalls associated with the use of TRPS1 IHC one should be aware of during dermatopathology practice.

## Figures and Tables

**Table 1:** Difference between different tumor types in terms of TRPS1 expression characteristics.

	Intensity Score				Proportion Score				H Score			
	0	1	2	3	Mean	Median	IQR	Range	Mean	Median	IQR	Range
AFX/PDS (N = 21)	1 (4.8%)	2 (9.5%)	6 (28.6%)	12 (57.1%)	80.0	90.0	15.0	0.0–100.0	209.5	240.0	125.0	0.0–300.0
Angiosarcoma (N = 20)	17 (85.0%)	1 (5.0%)	2 (10.0%)	0 (0.0%)	5.0	0.0	0.0	0.0–40.0	8.5	0.0	0.0	0.0–80.0
Dermatofibroma (N = 24)	0 (0.0%)	3 (12.5%)	21 (87.5%)	0 (0.0%)	66.7	70.0	20.0	5.0–95.0	131.2	140.0	45.0	20.0–190.0
DFSP (N = 22)	8 (36.4%)	10 (45.5%)	4 (18.2%)	0 (0.0%)	31.4	15.0	67.5	0.0–90.0	46.4	15.0	67.5	0.0–180.0
Kaposi sarcoma (N = 8)	6 (75.0%)	1 (12.5%)	1 (12.5%)	0 (0.0%)	1.9	0.0	1.2	0.0–10.0	3.1	0.0	1.2	0.0–20.0
Leiomyoma (N = 8)	0 (0.0%)	1 (12.5%)	6 (75.0%)	1 (12.5%)	75.0	85.0	15.0	20.0–90.0	158.8	170.0	30.0	20.0–270.0
Leiomyosarcoma (N = 8)	2 (25.0%)	2 (25.0%)	2 (25.0%)	2 (25.0%)	59.4	55.0	83.8	0.0–170.0	109.4	85.0	165.0	0.0–285.0
Melanoma (N = 9)	5 (55.6%)	4 (44.4%)	0 (0.0%)	0 (0.0%)	18.3	0.0	30.0	0.0–70.0	18.3	0.0	30.0	0.0–70.0
Neurofibroma (N = 22)	17 (77.3%)	4 (18.2%)	1 (4.5%)	0 (0.0%)	3.6	0.0	0.0	0.0–40.0	5.5	0.0	0.0	0.0–80.0
SSCC (N = 5)	0 (0.0%)	2 (40.0%)	1 (20.0%)	2 (40.0%)	63.0	60.0	30.0	15.0–90.0	138.0	60.0	210.0	30.0–270.0
Total (N = 147)	56 (38.1%)	30 (20.4%)	44 (29.9%)	17 (11.6%)	38.9	20.0	80.0	0.0–170.0	80.9	30.0	160.0	0.0–300.0
*p*-value	<0.001				<0.001 (<0.001 *)				<0.001 (<0.001 *)			

* *p*-value obtained from ANOVA, not recommended due to the violation of normality assumption. Abbreviations: IQR, interquartile range; AFX/PDS, atypical fibroxanthoma/pleomorphic dermal sarcoma; DFSP, dermatofibrosarcoma protuberans; SSCC, sarcomatoid squamous cell carcinoma.

**Table 2:** Difference between AFX/PDS group and angiosarcoma group in terms of TRPS1 expression characteristics.

	AFX/PDS (N = 21)	Angiosarcoma (N = 20)	Total (N = 41)	*p*-Value
Intensity Score				<0.001
0	1 (4.8%)	17 (85.0%)	18 (43.9%)	
1	2 (9.5%)	1 (5.0%)	3 (7.3%)	
2	6 (28.6%)	2 (10.0%)	8 (19.5%)	
3	12 (57.1%)	0 (0.0%)	12 (29.3%)	
Proportion Score				<0.001 (<0.001 *)
Mean	80.0	5.0	43.4	
Median	90.0	0.0	30.0	
IQR	15.0	0.0	90.0	
Range	0.0–100.0	0.0–40.0	0.0–100.0	
H score				<0.001 (<0.001 *)
Mean	209.5	8.5	111.5	
Median	240.0	0.0	50.0	
IQR	125.0	0.0	240.0	
Range	0.0–300.0	0.0–80.0	0.0–300.0	

* *p*-value obtained from two-sample *t*-test, not recommended due to the violation of normality assumption. Abbreviations: AFX/PDS, atypical fibroxanthoma/pleomorphic dermal sarcoma; IQR, interquartile range.

**Table 3:** Difference between AFX/PDS group and melanoma group in terms of TRPS1 expression characteristics.

	AFX/PDS (N = 21)	Melanoma (N = 9)	Total (N = 30)	*p*-Value
Intensity Score				<0.001
0	1 (4.8%)	5 (55.6%)	6 (20.0%)	
1	2 (9.5%)	4 (44.4%)	6 (20.0%)	
2	6 (28.6%)	0 (0.0%)	6 (20.0%)	
3	12 (57.1%)	0 (0.0%)	12 (40.0%)	
Proportion Score				<0.001 (<0.001 *)
Mean	80.0	18.3	61.5	
Median	90.0	0.0	80.0	
IQR	15.0	30.0	71.2	
Range	0.0–100.0	0.0–70.0	0.0–100.0	
H score				<0.001 (<0.001 *)
Mean	209.5	18.3	152.2	
Median	240.0	0.0	160.0	
IQR	125.0	30.0	247.5	
Range	0.0–300.0	0.0–70.0	0.0–300.0	

* *p*-value obtained from two-sample *t*-test, not recommended due to the violation of normality assumption. Abbreviations: AFX/PDS, atypical fibroxanthoma/pleomorphic dermal sarcoma; IQR, interquartile range.

## Data Availability

The data presented in this study are available on reasonable request from the corresponding author.

## Supplementary Materials

The following supporting information can be downloaded at: https://www.mdpi.com/article/10.3390/dermatopathology11030021/s1, Table S1: Difference between dermatofibroma group and DFSP group in terms of TRPS1 expression characteristics; Table S2: Difference between leiomyoma group and leiomyosarcoma group in terms of TRPS1 expression characteristics; Table S3: Difference between AFX/PDS group and leiomyosarcoma group in terms of TRPS1 expression characteristics; Table S4: Difference between AFX/PDS group and SSCC group in terms of TRPS1 expression characteristics.
