# Supplementary material for: TRPS1 Expression Is Frequently Seen in a Subset of Cutaneous Mesenchymal Neoplasms and Tumors of Uncertain Differentiation: A Potential Diagnostic Pitfall
Source: Dermatopathology (Basel). 2024 Jul 15;11(3):200–8. doi: 10.3390/dermatopathology11030021 (PMC11270280; doi:10.3390/dermatopathology11030021)
Supplement: Supplementary file 1 [file dermatopathology-11-00021-s001.zip › dermatopathology-3026440-supplementary.pdf]

Table S1. Difference between dermatofibroma group and DFSP group in terms of TRPS1 expression characteristics

	**Dermatofibroma (*N*=24)**	**DFSP (*N*=22)**	**Total (*N*=46)**	***p* Value**
Intensity Score	0 (0.0%)	8 (36.4%)	8 (17.4%)	<0.001
0	3 (12.5%)	10 (45.5%)	13 (28.3%)	
1	21 (87.5%)	4 (18.2%)	25 (54.3%)	
2				0.001 (<0.001 *)
Proportion Score	66.7	31.4	49.8	
Mean	70.0	15.0	65.0	
Median	20.0	67.5	67.5	
IQR	5.0–95.0	0.0–90.0	0.0–95.0	
Range				<0.001 (<0.001 *)
H score	131.2	46.4	90.7	
Mean	140.0	15.0	100.0	
Median	45.0	67.5	140.0	
IQR	20.0–190.0	0.0–180.0	0.0–190.0	
Range				

* *p*-value obtained from two-sample t-test, not recommended due to the violation of normality assumption. Abbreviations: DFSP, dermatofibrosarcoma protuberans; IQR, interquartile range.

Table S2. Difference between leiomyoma group and leiomyosarcoma group in terms of TRPS1 expression characteristics

	**Leiomyoma (*N*=8)**	**Leiomyosarcoma (*N*=8)**	**Total (*N*=16)**	***p* Value**
Intensity Score				0.298
0	0 (0.0%)	2 (25.0%)	2 (12.5%)	
1	1 (12.5%)	2 (25.0%)	3 (18.8%)	
2	6 (75.0%)	2 (25.0%)	8 (50.0%)	
3	1 (12.5%)	2 (25.0%)	3 (18.8%)	
Proportion Score				0.423 (0.498 *)
Mean	75.0	59.4	67.2	
Median	85.0	55.0	80.0	
IQR	15.0	83.8	47.5	
Range	20.0–90.0	0.0–170.0	0.0–170.0	
H score				0.205 (0.322 *)
Mean	158.8	109.4	134.1	
Median	170.0	85.0	150.0	
IQR	30.0	165.0	137.5	
Range	20.0–270.0	0.0–285.0	0.0–285.0	

* *p*-value obtained from two-sample t-test, not recommended due to the violation of normality assumption. Abbreviations: IQR, interquartile range.

Table S3. Difference between AFX/PDS group and leiomyosarcoma group in terms of TRPS1 expression characteristics

	AFX/PDS (*N*=21)	Leiomyosarcoma (*N*=8)	Total (*N*=29)	*p* Value
Intensity Score				0.170
0	1 (4.8%)	2 (25.0%)	3 (10.3%)	
1	2 (9.5%)	2 (25.0%)	4 (13.8%)	
2	6 (28.6%)	2 (25.0%)	8 (27.6%)	
3	12 (57.1%)	2 (25.0%)	14 (48.3%)	
Proportion Score				0.146 (0.196 *)
Mean	80.0	59.4	74.3	
Median	90.0	55.0	90.0	
IQR	15.0	83.8	35.0	
Range	0.0–100.0	0.0–170.0	0.0–170.0	
H score				**0.029** (0.024 *)
Mean	209.5	109.4	181.9	
Median	240.0	85.0	200.0	
IQR	125.0	165.0	165.0	
Range	0.0–300.0	0.0–285.0	0.0–300.0	

* *p*-value obtained from two-sample t-test, not recommended due to the violation of normality assumption. Abbreviations: AFX/PDS, atypical fibroxanthoma/pleomorphic dermal sarcoma; IQR, interquartile range.

Table S4. Difference between AFX/PDS group and SSCC group in terms of TRPS1 expression characteristics

	AFX/PDS (*N*=21)	SSCC (*N*=5)	Total (*N*=26)	*p* Value
Intensity Score				0.441
0	1 (4.8%)	0 (0.0%)	1 (3.8%)	
1	2 (9.5%)	2 (40.0%)	4 (15.4%)	
2	6 (28.6%)	1 (20.0%)	7 (26.9%)	
3	12 (57.1%)	2 (40.0%)	14 (53.8%)	
Proportion Score				0.121(0.220 *)
Mean	80.0	63.0	76.7	
Median	90.0	60.0	90.0	
IQR	15.0	30.0	22.5	
Range	0.0–100.0	15.0–90.0	0.0–100.0	
H score				0.201(0.161 *)
Mean	209.5	138.0	195.8	
Median	240.0	60.0	240.0	
IQR	125.0	210.0	136.2	
Range	0.0–300.0	30.0–270.0	0.0–300.0	

* *p*-value obtained from two-sample t-test, not recommended due to the violation of normality assumption. Abbreviations: AFX/PDS, atypical fibroxanthoma/pleomorphic dermal sarcoma; SSCC, sarcomatoid squamous cell carcinoma; IQR, interquartile range.
